# Development and Evaluation of the Wound Healing Effect of a Novel Topical Cream Formula Based on *Ginkgo biloba* Extract on Wounds in Diabetic Rats

**DOI:** 10.1155/2021/6474706

**Published:** 2021-10-13

**Authors:** Sana Bardaa, Khouloud Makni, Ons Boudaouara, Tarek Bardaa, Naourez Ktari, Selim Hachicha, Riadh Ben Salah, Rim Kallel, Zouheir Sahnoun, Sami Boufi

**Affiliations:** ^1^Laboratory of Pharmacology, Faculty of Medicine of Sfax, University of Sfax, Tunisia; ^2^GLOBUS Industry for Health Product, 2021 Sfax, Tunisia; ^3^Faculty of Science, LSME University of Sfax, BP1171, 3018 Sfax, Tunisia; ^4^Laboratory of Anatomopathology, CHU Habib Bourguiba, University of Sfax, 3029 Sfax, Tunisia; ^5^Department of Orthopedic Surgery and Traumatology, CHU Habib Bourguiba, University of Sfax, Tunisia; ^6^Laboratory of Enzyme Engineering and Microbiology, National School of Engineering of Sfax, University of Sfax, 3038 Sfax, Tunisia; ^7^Department of Life Sciences, Faculty of Science of Gabes, Omar Ibn Khattab Street, Gabes 6029, Tunisia; ^8^Laboratory of Microorganisms and Biomolecules (LMB), Centre of Biotechnology of Sfax, Road of Sidi Mansour Km 6, P.O. Box 1177, Sfax 3018, Tunisia

## Abstract

Despite advances in diabetes care, impaired diabetic wound healing remains a significant clinical problem. The present study was aimed at developing a novel cream based on *Ginkgo biloba* extract and investigating its wound healing effect on full-thickness wounds in diabetic rats. The topical formulated oil-in-water emulsion-based cream contains *Ginkgo biloba* aqueous extract in an amount of about 1% to 5% as an active agent. The prepared formula was subjected to physicochemical assessment and pharmacotechnical characterization. Eighteen alloxan-induced diabetic rats completing full-thickness excisional skin wounds were randomly divided into three groups topically treated with either a normal saline (control group), the reference drug (“Cytol Centella cream®”), and cream based on the *Ginkgo biloba* extract. The response to treatment was assessed by macroscopic, qualitative, and quantitative histopathological analysis. The prepared formula showed good physicochemical properties. The rheological behavior of the prepared cream followed a non-Newtonian pseudoplastic pattern at different storage temperatures. The cream, which is a macroemulsion with uniform size distribution, remained stable for 6 months. Skin tolerance studies confirmed the compatibility of the cream with the skin. During the experimental trial, the cream based on the *Ginkgo biloba*-treated group showed significant improvements over the control and reference groups for both general wound appearance and healing dynamics. This increased rate of closure of wounds in diabetic rats was associated with increased collagen synthesis. Our findings showed that the cream could be a promising and innovative topical treatment with *Ginkgo biloba* extract for the management of acute diabetic wounds.

## 1. Introduction

Diabetes mellitus, commonly known as diabetes, is one of the most serious and common metabolic chronic diseases characterized by a hyperglycemic state related to insulin deficiency. The main classes of diabetes include type 1 and type 2. Type 1 diabetes occurs due to the immune-mediated destruction of the pancreatic *β*-cells leading to insulin deficiency. Type 2 diabetes is a polygenic disorder that involves an impairment of insulin secretion related to insulin resistance [1]. Even though most diabetes complications are linked to its chronicity, one of the most severe complications of diabetes is impaired wound healing even in the early stage of the disease [2].

Cutaneous wound healing, which is triggered by tissue injury, is an intricate and highly coordinated process that includes four phases: (i) hemostasis; (ii) inflammation which is mediated by inflammatory cell recruitment, and growth factor and cytokine secretion; (iii) proliferation characterized by the formation of the granulation tissue and reepithelialization; and (iv) remodeling, in which granulation tissue is reorganized and the mature scar is formed [3, 4].

However, under hyperglycemic conditions, the natural healing process is ineffective to recover the damaged skin as all the stages of the wound healing process are disrupted and the wound repair is delayed [5]. Indeed, the cutaneous wound healing in acute diabetic patients is associated with abnormal and delayed inflammatory response characterized by the deficiencies in the number of neutrophils in the wound site [2]. This may increase the risk of bacterial infection that leads to a chronic nonhealing wound state posing a significant healthcare burden with consequences that include hospitalization and lower limb amputation. Due to the lack of preventive and control measures, the incidence of impaired healing process in diabetic patients is constantly increasing [6]. So, it is important to develop novel therapies to avoid any serious complications and accelerate wound healing process in the early diabetic stage.

In skin care therapy, cream formulas remain the gold-standard vehicles for topical drug delivery. Topical therapeutic efficacy is strongly related to skin conditions, physicochemical properties of the active substance, and the characteristics of the formula [7]. The understanding and selection of a suitable vehicle are of crucial importance, since it plays a major role on the formula's appearance, product performance, physical stability, and patient compliance [8]. Accordingly, this study is aimed at studying the characteristics of a newly developed formula based on *Ginkgo biloba* extract and at assessing its wound healing potential in diabetic rats.

*Ginkgo biloba* extract is derived from the leaves of the *Ginkgo biloba* tree (Ginkgoaceae), the world's most ancient tree species that is commonly found in China, Japan, and Korea [9]. *Ginkgo biloba* leaves and their extracts have been widely used in elderly populations and in traditional Chinese medicine for centuries [10]. Flavonoids (such as quercetin, kaempferol, and isorhamnetin) and terpenoids (including ginkgolide A, ginkgolide B, ginkgolide C, and bilobalide) as well as ginkgolic acids are the major classes of active compounds found in *Ginkgo biloba* extract [11, 12]. Although the exact mechanism remains unclear, these components exhibit a number of benefits including scavenging ROS, improving hemodynamics, and affecting platelet-activating factors and blood circulation. Indeed, *Ginkgo biloba* extracts are widely reported to treat and prevent a variety of ailments, including ischemic cerebral and heart diseases, diabetes, skin infection, cancer, and cognitive disorders [11, 13, 14]. However, the wound healing effect of *Ginkgo biloba* extract on diabetics is yet to be determined. Therefore, the present study was performed to evaluate the wound healing effect of a topical healing cream based on the *Ginkgo biloba* leaf aqueous extract in diabetic rats.

## 2. Materials and Methods

### 2.1. Materials

*Ginkgo biloba* leaf aqueous extract used in the current study is an extract of dried *Ginkgo biloba* leaves which was obtained from Greentech society (Phytelene EG 489). *Ginkgo biloba* extract is a standardized mixture containing 22-27% *Ginkgo* flavone glycosides represented by myricetin, quercetin, kaempferol, and isorhamnetin and 5-7% terpene lactones (ginkgolide A, B, and C and bilobalides) and less than 5 mg/kg (5 ppm) ginkgolic acids.

Alloxan (CAS Number: 2244-11-3) was purchased from Sigma-Aldrich; Merck KGaA.

“Cytol Centella cream®” is a synthetic oil in water emulsion drug. The active principle of this drug is based on a natural titrated extract of *Centella asiatica* used as a therapeutic agent in wound healing that helps to restore damaged skin.

### 2.2. Animals

Adult healthy male Wistar rats aged between 10 and 12 weeks and weighing 160 ± 22 g were obtained from the Society of Pharmaceutical Industries of Tunisia (SIPHAT). All animals were housed in an environmentally controlled room in accordance with the Guide for Care and Use of Laboratory Animals at constant temperature (22 ± 1°C) with a 12/12 h light/dark cycle. All rats had free access to water and standard laboratory food. The experiments were designed following animal welfare guidelines and the three R's principles.

### 2.3. Galenic Formulation

The cream was prepared in the GLOBUS Industry for Health Product, a subsidiary company of the pharmaceutical industry SIMED in the framework of a research project funded by the European Union. The concentrations of excipients were in accordance with the *Handbook of Pharmaceutical Excipients*.

### 2.4. Cream Composition

The topical formula used in this study consisted of the following: (1) an active agent comprising *Ginkgo biloba* aqueous extract in an amount of about 1% to 5% (*w*/*w*); (2) an emollient caprylic/capric triglyceride in an amount of about 3% to 15% (*w*/*w*); (3) a natural viscosity increasing agent in an amount of about 2% to 10% (*w*/*w*); (4) a water-in-oil emulsifying agent in an amount of about 0.5% to 10% (*w*/*w*); (5) a thickening agent sodium polyacrylate in an amount of about 0.1% to 2%; (6) a moistening agent glycerol in an amount of about 2% to 10%; and (7) an antimicrobial preservative in an amount of about 0.05% to 1% (*w*/*w*) (Table 1).

### 2.5. Cream Preparation

The cream was formulated following the method described by Chen et al. [15] with suitable modifications. The aqueous and oily phases were heated separately to ~75°C. The oily phase was then added to the aqueous phase, and the mixture was mixed at high speed with a disperser (IKA T18 Basic ULTRA-TURRAX). The mixture was then cooled with constant mixing to form a semisolid base. When the temperature of the emulsion reached 40°C, the active agent and the antimicrobial preservative were dispersed with continuous stirring.

### 2.6. Characterization of the Cream

#### 2.6.1. Physical Properties of the Cream

The prepared formula was examined for its physical properties of color, odor, texture, homogeneity, spreadability, and skin feel. The formula was tested for homogeneity by visual appearance and touch. The consistency and presence of coarse particles were used to evaluate the texture and homogeneity of the formula [15]. To ensure the stability of the quality of the cream, three transparent cream jars were left at room temperature in the laboratory and examined after 1 week, 1 month, 3 months, and 6 months.

#### 2.6.2. pH Determination

One gram of the prepared cream was dispersed in 25 mL of purified water. The pH of the mixture was measured at ambient temperature using a digital pH meter (Thermo Scientific Expert pH). The assay was carried out in triplicate for the formulated cream, and the average value was calculated.

#### 2.6.3. Optical Microscopy Analysis

The prepared emulsion was examined under a microscope (Motic AE2000) fitted with a digital camera. A drop of the cream was smeared onto a glass microscope slide and made as thin as possible by covering and pressing down with a coverslip [16]. A 200x magnification lens was used to view the sample.

#### 2.6.4. Particle Sizing Distribution (PSD)

Droplet-size distributions of the emulsion were determined with a Mastersizer 2000 (Malvern Instruments, Malvern, UK). Measurements were made on emulsions diluted to about 5% by addition of water. The measurements were systematically carried out in triplicate.

The diameter size was expressed as the volume-surface mean diameter (D32) calculated according to Eq. (1)

(1) $$\begin{array}{c} D_{32}=\frac{\sum n_{i}d_{i}^{3}}{\sum n_{i}d_{i}^{2}}, \end{array}$$

where *n*_*i*_ is the droplet number with diameter *d*_*i*_.

#### 2.6.5. Rheological Measurements

The rheological measurements were performed on a Kinexus Pro+rheometer (Malvern Instruments, UK) using a plate-plate geometry with a diameter of 25 mm with a rough surface to prevent slippage to the wall. The temperature was kept constant at 25°C using the Peltier heating system. Under dynamic mode, the linear domain was first determined by a sweep of the storage modulus *G*′ and loss modulus *G*^″^ vs. strain from 0.01 to 100%. Then, a frequency sweep at a fixed strain in the elastic linear domain was made. In shear rate measurement, a cone plate geometry was used (cone angle, 2°; diameter, 20 mm; truncation, 56 *μ*m) with a ramp from 0.1 to 100 s^−1^.

#### 2.6.6. Acute Dermal Toxicity

To assess the acute dermal toxicity of the prepared cream, six Wistar rats with intact skin were used. The back of rats was carefully shaved and applied with 100 mg of the prepared cream. The treated area was then covered with a cotton bandage for 24 hrs. The animals were later assessed for signs of acute toxicity [17].

### 2.7. Wound Healing Potential of the *Ginkgo biloba*-Based Cream

#### 2.7.1. Alloxan-Induced Diabetes

To assess the wound healing effect of the prepared cream in vivo, 18 adult Wistar rats weighing 160 ± 22 g were selected for use. The rats fasted overnight and were subjected to a single intraperitoneal injection of alloxan at a concentration of 130 mg/kg body weight, dissolved in 0.9% saline according to Martins et al. [18]. The injected rats immediately received 20% glucose solution for 6 hours to prevent fatal hypoglycemia that often follows alloxan treatment. For the next 24 h, the rats were then given a 5% glucose solution as beverage. The measurement of blood glucose level was performed a period of one week after alloxan injection using a glucometer via the tail vein. Animals with a fasting blood glucose concentration above 2 g/L were considered diabetic.

#### 2.7.2. Model of Excisional Wound

The eighteen diabetic rats were anesthetized by intramuscular injection of 50 mg/kg of ketamine, shaved on the back with an electric clipper, and disinfected with 70% (*v*/*v*) ethanol. A metal punch was used to demarcate an area of skin for removal. The rats were then subjected to identical wounds; a full-thickness open excision oval wound (2 cm × 1 cm) was made by removing a patch of skin with a pair of surgical scissors.

#### 2.7.3. Experimental Protocol

After wound induction, the diabetic animals were randomly divided into three equal groups as follows:

Group 1: the wounded skin group was just cleaned by a saline solution assigned as control.

Group 2: the wounded skin group was treated with 0.13 mg/mm^2^ of a reference drug named “Cytol Centella cream®” which is a synthetic cream drug (CCC group).

Groups 3: wounded skin groups were treated with 0.13 mg/mm^2^ of the prepared cream based on *Ginkgo biloba* aqueous extract (GBAEC group).

The topical treatments and bandaging with sterilized dressing film were carried out every 2 days starting from the wound induction until the first group was completely healed.

#### 2.7.4. Blood Glucose Measurement

The blood glucose levels of the treated rats were checked on the final day of the trial.

#### 2.7.5. Measurement of Wound Area

The changes in wound edge diameter were measured (in mm) by tracing the wound boundaries on a transparent paper every two days before treatment application. The wound edge contraction was expressed as the decrease of the original wound size percentage.

The percentage of wound contraction was calculated by using the following equation: Percentage of wound contraction = [(Initial wound burn size–specific day wound size)/Initial wound burn size] × 100 [19].

All experimental analyses were performed in a blinded manner.

#### 2.7.6. Histology

After the 13^th^ day, the rats were euthanized and the autopsy samples were removed from the middle of the injured skin of the rat in all the groups. The samples were fixed in 10% neutral buffered formalin solution for a week. After dehydration, the specimens were embedded in paraffin beeswax and tissue blocks were sectioned at 5-micron thickness using a sledge microtome. Prepared tissue sections were then stained by hematoxylin and eosin stain for microscopic examination. Digital photomicrographs were captured at representative locations, and the tissue scar was analysed for the extent of dermis and epidermis formation and regeneration. A semiquantitative method was carried out to assess the following histological processes and structures: reepithelization, inflammatory cells, and new collagen. Masson's trichrome staining was used to detect collagen fibers. Sections were evaluated according to the scale: 0, 1, 2, 3, and 4 by two independent observers [20]. The mean value was used for statistical comparison. The grading scale is as follows: 0 absent, 1 slight, 2 moderate, 3 marked, and 4 very marked.

#### 2.7.7. Statistical Analysis

Data were reported as the mean ± standard deviation (S.D). Statistical analyses were performed with one-way analysis of variance (ANOVA) followed by Tukey and Fisher tests. The different rat groups were compared on the basis of the wound area contraction and the semiquantitative preestablished histological scores. A value of *p* < 0.05 was considered significant.

## 3. Results

### 3.1. Physicochemical Properties

The physicochemical properties, regarding physical appearance, color, odor, texture, homogeneity, phase separation, immediate skin feel, and pH of the prepared cream kept at different storage conditions, are shown in Table 2.

The prepared cream showed good macroscopic quality characterized by a milky aspect, homogeneous appearance, semisolid consistency, and easy spreadability. Its application on the skin gave a moisturizing, nonsticky, and nongreasy touch. The pH was slightly acidic (5.43) and was regulated to stay within the narrow range of 6.36. The prepared cream was stable at different storage conditions. It did not show any visual degradation during 6 months. The cream was able to maintain its physicochemical characteristics at ambient temperature.

### 3.2. Optical Microscopy Analysis

**Figure 1:**
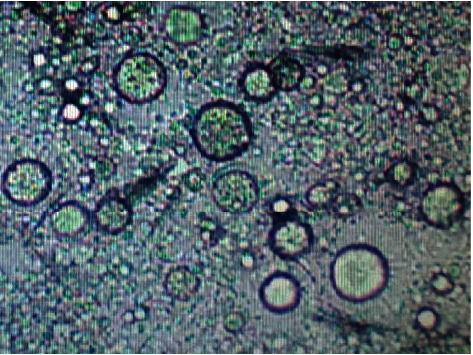
Microphotograph of the prepared cream based on *Ginkgo biloba* extract magnification: 200x.

The morphology of the prepared cream is shown in Figure 1. The prepared cream is an oil-in-water emulsion. The microphotograph of the prepared cream clear showed heterogeneous phases. The oil was dispersed in the water phase in the form of large milky droplets.

### 3.3. Colloidal Properties of the Emulsion

#### 3.3.1. Droplet Size Distribution

**Figure 2:**
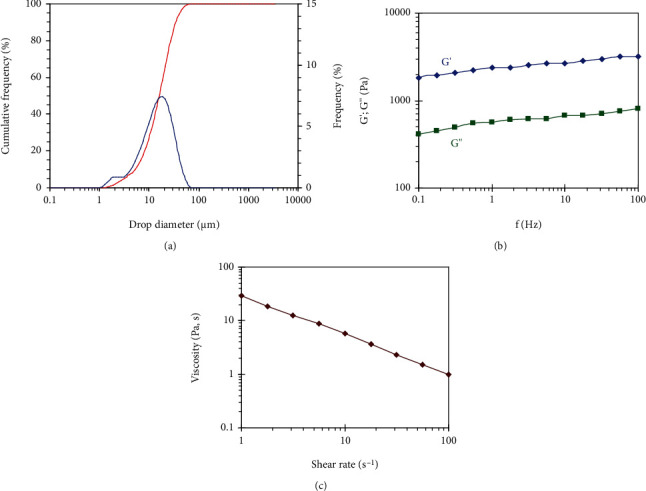
(a) Droplet size distributions of the emulsion, (b) *G*′ and *G*^″^ as a function of frequency (Hz) in the linear domain, and (c) viscosity as a function of shear rate of the emulsion.

The droplet size distribution of the emulsion (Figure 2(a)), assessed by the light diffraction method, indicated a quasi-Gaussian distribution ranging from 2 to 100 *μ*m, with a D50 (diameter D50 at 50% of cumulative volume), was around 15 *μ*m.

#### 3.3.2. Rheological Properties

The rheological properties of the emulsions were investigated in the linear by oscillatory sweep measurements of the storage modulus (*G*′) and loss modulus (*G*^″^) as a function of frequency (*f*) (Figure 2(b)) and in the nonlinear domain by measuring the steady-state viscosity vs. shear rate (Figure 2(c)). In the domain of linear elasticity (i.e., in the range of strain where *G*′ and *G*^″^ are independent of the strain) (Figure 2(b)), *G*′ and *G*^″^ were nearly frequency-independent, with *G*′ being higher than *G*^″^, confirming a gel-like behavior of the emulsion. Based on the results of the rheological behavior of the cream, the viscosity decreased as the shear rate increased.

#### 3.3.3. Acute Dermal Toxicity

Neither erythema nor any kind of allergic reaction was noted in the dorsal back following dermal application during 24 hours.

#### 3.3.4. Blood Sugar Level

The mean sugar level for all rats was in the order of 2.5 ± 0.3 g/L; however, it was only 1 ± 0.2 g/L for normal rats. The blood glucose levels in the diabetic rats were not changed by topical treatment of the wound using either reference or *Ginkgo biloba* creams.

### 3.4. Assessment of the Healing Effect

#### 3.4.1. Macroscopic Qualitative Analysis

**Figure 3:**
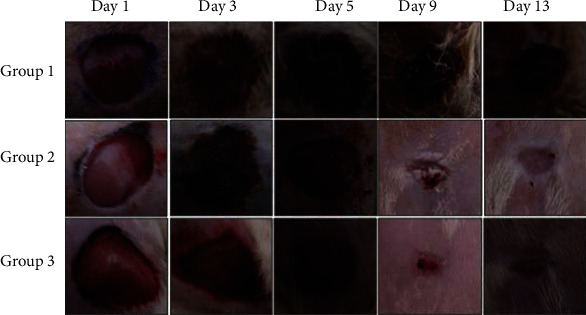
Representative photographs of macroscopic assessment of wounds for the three studied groups on day 1, day 3, day 5, day 9, and day 13. Group 1: diabetic rats were treated with saline solution (control group); Group 2: diabetic rats were treated with reference drug “Cytol Centella cream®”; Group 3: diabetic rats were treated with the cream based on *Ginkgo biloba* extract.

Wounds were regularly photographed and evaluated from day 1 until the first group is completely healed. The healing process lasted 13 days in the GBAEC group. Photographs of the wounds of a representative rat from each group are shown in Figure 3.

On the first day of injury induction, all wounds had a similar appearance. During the experiment, remarkable differences in wound area and morphology of different groups can be observed. From the third day, a scab formed by necrotic tissue remnants was present in the control and CCC groups. The scab persisted in the control group until the end of the experiment. The scab in the reference group was thinner than in the control group. The clinical signs of inflammation (redness and swelling) were greater in the control and the CCC groups when compared to the GBAEC group. From the fifth day, a very thin scab appeared in the GBAEC group. The scab started to fall off by the ninth day of the trial, and the epidermis was completely developed.

In the GBAEC group, the size of scar tissue was also smaller and better than in the CCC group. In this group, healing was much faster than that in the control and CCC groups. Reepithelialization was faster in the GBAEC group when compared with other groups.

#### 3.4.2. Macroscopic Quantitative Analysis

**Figure 4:**
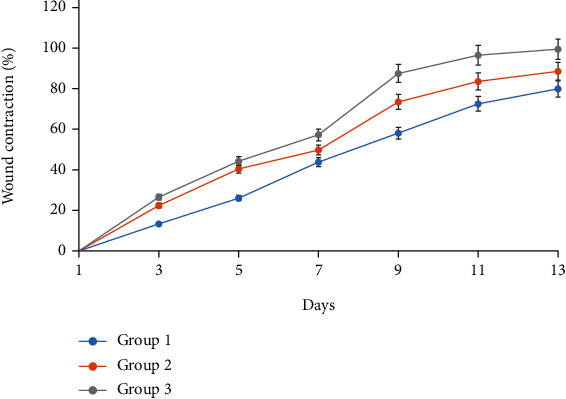
Evolution of the percentage of wound contraction for the three studied groups. Group 1: diabetic rats were treated with saline solution (control group); Group 2: diabetic rats were treated with reference drug “Cytol Centella cream®”; Group 3: diabetic rats were treated with the cream based on *Ginkgo biloba* extract.

The average wound surfaces of the three studied groups are presented in Table 3.  Figure 4 illustrates the average percentages of contraction of wound surfaces on the 1^st^, 3^rd^, 5^th^, 7^th^, 10^th^, 11^th^, and 13^th^ days of the study.

During the study, the GBAEC group was clearly distinguished from the other groups by a faster healing.

At day 13 postinjury, the closure of the diabetic wounds treated with GBAEC (100%) was significantly faster than those of the control (80.28%) and CCC groups (89.07%).

#### 3.4.3. Histology

**Figure 5:**
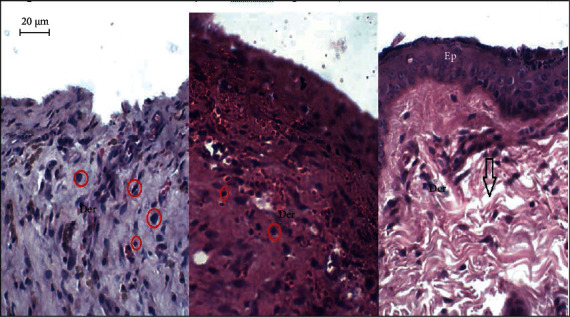
Hematoxylin and eosin- (H&E-) stained sections (×200) from wounded area of diabetic rats 13 days after wound induction. Representative histological section of an untreated wound control; (b) Histological section of a scar area of a reference rat treated with “Cytol Centella cream®”; (c) Histological section of a scar area of a rat treated with the cream based on Ginkgo biloba extract. Der: dermis; Ep: epidermis; arrow down: blood vessels; red circle: inflammatory cells.

**Figure 6:**
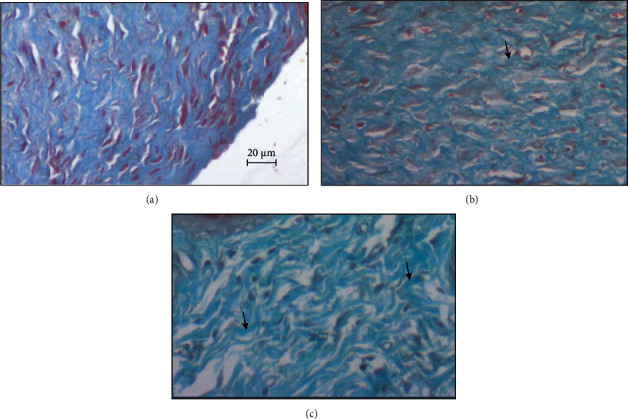
Masson's trichrome stained sections (×200) from wounded area of diabetic rats 13 days after wound induction showing arrangement of dermal collagen fibers. (a) Representative histological section of an untreated wound control; (b) histological section of a scar area of a reference rat treated with “Cytol Centella cream®”; (c) histological section of a scar area of a rat treated with the cream based on *Ginkgo biloba* extract.

The histopathological studies were carried out to assess the wound healing process in different studied groups. The hematoxylin and eosin- and Masson's trichrome-stained skin tissue sections at 200x magnification are shown in Figures 5 and 6, respectively.

**Figure 7:**
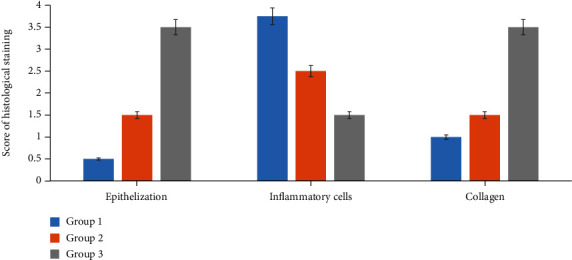
Semiquantitative analysis of histological structures at day 13 post wound injury. Group 1: diabetic rats were treated with saline solution (control group); Group 2: diabetic rats were treated with reference drug “Cytol Centella cream®”; Group 3: diabetic rats were treated with the cream based on *Ginkgo biloba* extract.

On day 13, the skin autopsies of the GBAEC group showed a continuous and complete epidermal layer that covered the entire area of the wound. In this group, the regenerated dermis was thicker than that of the control and CCC groups. In contrast, the skin autopsies of the CCC group were partially covered with epidermis. The histological scoring of epithelialization in the GBAEC group was significantly higher than those assessed in the control and CCC groups (*p* < 0.05) (Figure 7). In particular, the GBAEC group showed better collagen alignment on the regenerated skin tissues compared with the other groups as revealed by Masson's trichrome staining (Figure 6). The tissue of the CCC group was significantly infiltrated with inflammatory cells when compared to the other groups. As compared to the CCC and reference groups, the GBAEC group showed that the tissue regeneration was significantly accelerated and the inflammatory phase was in its final phase.

## 4. Discussion

Diabetes mellitus is a chronic hyperglycaemic disorder that leads to delaying wound closure. The application of phytochemicals could be a beneficial approach to improve the wound healing in diabetes [21].

In this context, our study is aimed at developing a topical healing cream based on *Ginkgo biloba* leaf aqueous extract and at evaluating its healing effect in diabetic rats in comparison with a reference product, “Cytol Centella cream®.”

The prepared cream is stable and showed good physicochemical properties. During the experimental trial, the GBAEC group showed significant improvements over the control and CCC groups for both general wound appearance and healing dynamics.

The study of the quality of the cream focused on the macroscopic characteristics of the formula, homogeneity, pH, and dynamic viscosity. The prepared cream is a semisolid emulsion that exhibited good macroscopic parameters and perfect stability. During 6 months, the prepared cream maintained its intended physical and chemical qualities, as well as functionality when stored under appropriate conditions. According to Medina-Torres et al. [22], the sodium polyacrylate used in our formula is a very good stabilizer which increases the effectiveness of the functional substances in the product. The pH value of the cream after formulation was around 5.43. This result is probably related to the acidic nature of the *Ginkgo biloba* leaf aqueous extract. The pH of the cream was then regulated to 6.36. Such a pH stimulates the activity of immune cells and protects the wound from invading microorganisms [23, 24]. This pH would also be in favor of chemical compatibility with the user's skin. This tolerance has already been confirmed by the allergy skin test on rats which showed a total absence of irritation and allergenic reactions. The allergy to a substance is a state of hypersensitivity reaction of the skin which is an immune response to an antigen that appears so excessive or inappropriate and is also manifested as erythema and edema. The absence of these reactions reflects the nonirritant status of the cream based on *Ginkgo biloba* extract [25].

The centrifugation test is considered as an accelerated stability test for predicting the long-term stability of the emulsion. This assay uses centrifugal force to separate two substances with different densities [26]. The prepared formula exhibited sufficient resistance to the destabilization phenomenon at ambient temperature. This might be attributed to the strong interfacial film between the aqueous and oil phases [27]. Indeed, the emulsifying agent used in our formula led to a strong repulsion of the oil droplets and prevents them from aggregation, flocculation, and coalescence over time.

The droplet size of the topical formula is an important characteristic for the physical stability of topical products [28]. Droplet size analysis in our study showed large, white, oily droplets trapped in the middle of the aqueous phase with a size range from 2 to 100 *μ*m with a D50 around 15 *μ*m (diameter D50 at 50% of cumulative volume). This distribution is typical of O/W emulsion cream stabilized with a conventional nonionic surfactant which promotes tissue regeneration and enhances skin penetration of the active ingredient [29, 30]. This type of emulsion system is known as a macroemulsion as the dispersed droplets are bigger than 0.1 *μ*m. The majority of emulsions belong to this category. This type of emulsion is kinetically stable but usually thermodynamically unstable as the two phases tend to break down and separate (flocculation or coalesce) due to the reduction in interfacial energies over time [31]. However, the powerful emulsifying agent used in our formula led to a strong repulsion of the oil droplets and eliminated their rate of coalescence [32]. The diameters are distributed in a single peak; hence, the distribution is unimodal [33]. The absence of air bubbles in the cream, clearly observed under a microscope, further guarantees its stability.

Rheology deals with the manufacturing and application of semisolid topical formulas. The rheological properties are crucial in determining several technological aspects of the product especially the physical stability of the formula during the shelf life and its spreadability [34]. Indeed, the rheological studies carried out on semisolid systems describe the relation between stress and deformation, mainly in systems for topical application [35]. The rheological properties of the emulsion confirmed a gel-like behavior of the emulsion, meaning that the emulsion exhibited a cream aspect at rest. The gel-like property of the emulsion is indicative of the generation of networks resulting from the presence of an acrylic thickener in the emulsion formula [36].

The viscosity test showed that the viscosity decreased with increasing shear rate which is typical of a shear-thinning behavior for pseudoplastic materials. This effect is presumably due to the disruption of the network generated by the thickening agent and to the alignment of oil droplets under the effect of applied shear rate which results in less interaction and a decrease in the viscosity. This rheological behavior ensures easy spreading on the skin [34]. The characteristics of this shear-thinning behavior provide pertinent information on the flow properties of the product which affect, for example, the way in which the technician must pump the product in the factory or the sensory impression perceived by the consumer when using the product [37].

The in vivo study testing the wound healing effect of the cream based on *Ginkgo biloba* extract was carried out on the model of a diabetic rat induced by alloxan. The use of the experimentally induced diabetes mellitus model by alloxan is one of the most used and convenient methods for studying and screening new drugs and new therapeutic modalities [38]. Alloxan, which is a chemically unstable organic compound, is a diabetogenic agent widely used to induce type 1 diabetes mellitus. Animals develop type 1 diabetes when they receive a varying dose of alloxan ranging from 80 to 140 mg/kg [39]. The concentration required to induce this type of diabetes depends closely on the animal species and the route of administration. In our study, the intraperitoneal injection of alloxan induced diabetic hyperglycemia (2 g/L ≤ blood glucose) in rats a week post its administration. Previous studies have also reported the diabetogenicity of alloxan following its administration to experimental rats [2, 21]. Alloxan is well-known to destroy the beta cells of the pancreas and causes hyperglycemia in rats [40]; also, it is selectively toxic for pancreatic *β*-cells of the islets of Langerhans by induction of necrosis [41]. Alloxan is a cytotoxic glucose analog which presents a molecular shape analogy with glucose [42] and has two distinct pathological effects: selective inhibition of glucose-induced insulin secretion through specific inhibition of glucokinase and generation of free radicals [43]. The mechanism for alloxan-induced diabetes is that free radicals generated by alloxan initiate damage that leads to destruction of pancreatic *β*-cells of the islets of Langerhans. It was reported that alloxan is toxic to pancreatic cells as it especially accumulates in the beta cells as glucose analogs. Thus, alloxan infiltrated the pancreatic *β*-cells through the GLUT2 transporter [44]. In the *β*-cell cytosol, alloxan is reduced to dialuric acid and the reduction of alloxan leads to generate reactive oxygen species (ROS) [40]. These oxidative agents provoke necrosis in pancreatic *β*-cells which leads to diabetes mellitus type 1 [45]. Alloxan also selectively inhibits glucose-induced insulin secretion through its ability to inhibit many functional enzymes such as glucokinase, the most sensitive enzyme in the *β*-cell that controls insulin secretion. This glycoregulatory disorder activates certain pathways for the generation of free radicals in oxygen. The oxidative stress thus generated could constitute the final mechanism at the origin of the oxidative complications associated with hyperinsulinemia [40].

The wounds in the GBAEC group contracted more rapidly and significantly than those in the control and CCC groups. This finding agrees with many studies in which the diabetic wounds of the untreated group always showed incomplete wound contraction than those treated with the tested healing principle [46–48].

Complete healing was observed after 13 days in the rats of the GBAEC group. This healing time is shorter than that observed by Pirbalouti et al. [46] (18 days) for wounds treated with *Punica granatum* with an initial surface area of 150 mm^2^ only against 160 mm^2^ for the wounds in our study. *Ginkgo biloba* extract seems to accelerate the healing process thanks to its pharmacological properties linked to its bioactive compounds (flavonoids, terpene trilactones, and ginkgolic acids). Indeed, the antioxidant, anti-inflammatory, and vasoregulatory properties of *Ginkgo biloba* extract already reported by certain authors [49–51] are likely to constitute factors for activating healing.

In acute diabetes, the inability of wounds to heal is related to aberrations of the wound healing process. Indeed, according to Komesu et al. [2], acute diabetes can be responsible for deficiencies in defense cells (low density of neutrophils) and in repair tissue failures. In our study, we observed an impaired wound repair in diabetic untreated rats associated with a prolonged inflammatory phase and reduced collagen production. Collagen is an essential building block of the skin that promotes wound healing. Thus, when wound repair is defective, stimulating the synthesis of the collagen would be beneficial for promoting wound healing. These results suggest that topical application of the cream based on *Ginkgo biloba* extract stimulated the deposition of collagen and thereby promoted the wound healing. Moreover, the *Ginkgo biloba*-based cream seems to enhance wound healing in diabetics by promoting the expression of vascular endothelial growth factor (VEGF). Indeed, according to Sun et al. [52], the extract of *Ginkgo biloba* promoted the expression of VEGF. VEGF is an endogenous stimulator of angiogenesis, and its receptors are upregulated during wound healing [53, 54]. Angiogenesis increases the delivery of oxygen and other nutrients that are necessary for local collagen synthesis [55].

## 5. Conclusions

On the basis of the results of our study, the cream based on *Ginkgo biloba* extract seemed to provide a significant advancement in wound repair when compared with the control and reference groups. To the best of our knowledge, the results of this study show for the first time the efficacy of *Ginkgo biloba*-based cream treatment on wound healing in diabetic animals. Hence, the results open the way to future clinical applications. However, further biochemical investigations are required to determine the accurate mechanism of the wound healing effect of the cream.

## Figures and Tables

**Table 1:** Cream composition.

Aqueous phase	Purified water	60-80%
	Sodium polyacrylate	0.1-2%
	Glycerol	2-10%
Oily phase	Water-in-oil emulsifying agent	0.5-10%
	Caprylic/capric triglyceride	3-15%
	Viscosity increasing agent	2-10%
Active agent	Ginkgo biloba aqueous extract	1-5%
Antimicrobial preservative	Antimicrobial preservative	0.05-1%

**Table 2:** Physicochemical properties of the prepared cream based on *Ginkgo biloba* extract at different storage conditions.

Control tests	Freshly formulated cream at ambient temperature	Storage at ambient temperature for 1 week, 1 month, 3 months, and 6 months	Storage at 50°C for a week
Appearance	Homogenous	Homogenous	Homogenous
Color	White	White	White
Odor	Characteristic odor of Ginkgo biloba extract	Characteristic odor of Ginkgo biloba extract	Characteristic odor of Ginkgo biloba extract
Consistency	Semisolid	Semisolid	Fluid
Texture	Smooth	Smooth	Smooth
Homogeneity phase separation Under centrifugal forces	Homogeneous, no phase separation	Homogeneous, no phase separation	Homogeneous, no phase separation
Spreadability	Easy	Easy	—
Penetration	The cream penetrates with light message	The cream penetrates with light message	—
Immediate skin feel	Moisturizing, light, no grittiness, nongreasy, nonsticky	Moisturizing, light, no grittiness, nongreasy, nonsticky	—
pH	pH = 5.43 stabilized with triethanolamine to obtain pH = 6.36	pH = 6.36	pH = 6.36

**Table 3:** Measurement of mean wound surface area (cm^2^) of the three studied groups.

Days	Day 1	Day 3	Day 5	Day 7	Day 10	Day 11	Day 13
Group 1	1.615 ± 0.051^a^	1.400 ± 0.063^a^	1.193 ± 0.155^a^	0.905 ± 0.129^a^	0.673 ± 0.172^a^	0.438 ± 0.129^a^	0.318 ± 0.145^a^
Group 2	1.631 ± 0.123^a^	1.246 ± 0.124^b^	0.968 ± 0.177^b^	0.816 ± 0.117^b^	0.426 ± 0.107^b^	0.260 ± 0.054^b^	0.178 ± 0.035^b^
Group 3	1.603 ± 0.040^a^	1.176 ± 0.218^b^	0.890 ± 0.144^b^	0.681 ± 0.158^b^	0.193 ± 0.480^c^	0.048 ± 0.075^c^	0.00 ± 0.00^c^

Values are given as mean ± SD (*n* = 6/group). Data with different letters for each column represent significant difference at *p* < 0.05. Group 1: diabetic rats were treated with saline solution (control group); Group 2: diabetic rats were treated with reference drug “Cytol Centella cream®”; Group 3: diabetic rats were treated with the cream based on *Ginkgo biloba* extract.

## Data Availability

The data used to support the findings of this study are included within the article.
